# Serum Proteomic Study of Women With Obsessive-Compulsive Disorder, Washing Subtype

**DOI:** 10.32598/bcn.9.5.337

**Published:** 2018-09-01

**Authors:** Mona Zamanian-Azodi, Mostafa Rezaei-Tavirani, Mohammad Mahboubi, Mohsen Hamidpour, Majid Rezaei Tavirani, Mostafa Hamdieh, Mohammad Rostami-Nejad, Naser Nejadi, Mohammad Kamran Derakhshan

**Affiliations:** 1. National Elites Foundation, Tehran, Iran.; 2. Proteomics Research Center, School of Paramedical Sciences, Shahid Beheshti University of Medical Sciences, Tehran, Iran.; 3. Department of Public Health, School of Medical Sciences, Abadan University of Medical Sciences, Abadan, Iran.; 4. Department of Surgery, School of Medicine, Iran University of Medical Sciences, Tehran, Iran.; 5. Department of Psychosomatic, Taleghani Hospital, Faculty of Medicine, Shahid Beheshti University of Medical Sciences, Tehran, Iran.; 6. Research Institute for Gastroenterology and Liver Diseases, Shahid Beheshti University of Medical Sciences, Tehran, Iran.; 7. Department of Clinical Biochemistry, Faculty of Medical Science, Tarbiat Modares University, Tehran, Iran.

**Keywords:** Obsessive Compulsive Disorder, Biomarkers, Proteomics, Protein interaction maps

## Abstract

**Introduction:**

Many genetic studies are conducted on Obsessive-Compulsive Disorder (OCD). however, a high-throughput examination of proteome profile of this severe disease has not been performed yet.

**Methods:**

Here, the proteomic study of OCD patients’ serum samples was conducted by the application of Two-Dimensional Electrophoresis (2DE) followed by Mass Spectrometry (MALDI-TOF-TOF).

**Results:**

A total of 240 protein spots were detected and among them, five significant differentially expressed protein spots with the fold change of ≥1.5 were considered for further evaluations. These proteins include IGKC, GC, HPX, and two isoforms of HP. While IGKC and HP show down-regulation, GC and HPX indicate up-regulation. Moreover, a validation study of overall HP levels in patients’ serum via nephelometric quantification confirmed the lower levels of this protein in the serum of OCD patients. Additionally, enrichment analysis and validation test revealed that inflammation is one of most dominant processes in OCD.

**Conclusion:**

It is suggested that these candidate proteins and their underlying processes (especially, inflammation) may be linked to OCD pathophysiology and can promise a clinical use after extensive validation studies.

## Highlights

- Proteins of IGKC and HP with two isoforms are down-regulated in Obsessive Compulsive Disorder (OCD) whereas HPX and GC proteins are up-regulated in this disorder.
- HP showed as a promising biomarker for OCD.
- Inflammation process is highly linked to the OCD risk.
- This research is a complementary study of the previous investigation “Serum Proteomic Profiling of Obsessive-Compulsive Disorder, Washing Subtype: A Preliminary Study”.

## Plain Language Summary

Obsessive-Compulsive Disorder (OCD) is a mental condition that imposes hardship in patients’ life. It has different types, one of the most important ones in women is washing rituals. One of the promising ways to understand this disorder is by molecular study. In this regard, proteomic research of proteins with differential expression in OCD has been conducted and five protein spots, including IGKC, GC, HPX, and HP (two isoforms) have been identified in the serum. In addition, inflammation as one of the key underlying processes of these proteins except for GC, may play fundamental role in OCD risk. These molecules and their significant process can be used as detectors for disorder diagnosis and treatment goals.

## 1. Introduction

Obsessive-Compulsive Disorder (OCD) as a complex and debilitating mental condition, has about 2% to 3% lifetime prevalence around the world. The disorder is typical with unpleasant thoughts and compulsive behavior (Taylor, 2013) that leads to dimensional life impairments (Sinopoli, Burton, Kronenberg, & Arnold, 2017). The etiology of OCD has still been remained inconclusive. Treatment options for OCD are available; however, not promising for all cases (Zamanian-Azodi et al., 2015) due to the heterogeneity of the disorder and limited pharmacotherapy approaches that are mostly designed for specific neurotransmitters (Zamanian-Azodi et al., 2017).

This fact entails the evaluation of other targets to better elucidate the underlying mechanisms of the disorder. In fact, OCD is considered 40% to 45% heritable. That is, the first-degree family of the patients with OCD has 4 to 10 times higher susceptibility to the disease (Fan et al., 2015). Evidently, a complex combination of genetic and environmental factors is related to the disorder etiology (Taylor, 2013) Interaction between these factors can result in different phenotypes known as subtypes (Jaffe et al., 2014). Molecular research can be beneficial in this regard; still, most of the studies on OCD are focused on genetic concept and the related polymorphisms as well as genome wide association studies (Mattheisen et al., 2015).

Meanwhile, application of high throughput methods can be helpful to identify other molecular signatures especially proteins as the functional part of the organisms (Karbalaei, Piran, Rezaei Tavirani, Asadzadeh Aghdaei, & Heidari, 2017). Proteomics has been proved to be promising for psychiatric disorders. Many candidate biomarkers have been purposed for schizophrenia, depression, and bipolarity (Föcking et al., 2016; Korth et al., 2017; Mendez-David et al., 2017).

One of the worthy human sources for proteome evaluation is serum. Many protein biomarkers can be detected through serum analysis, as it is easily provided and manageable. About thousands of secreted or leaked proteins from normal or damaged cells and tissues are circulating in blood (Tang, Beer, & Speicher, 2011). On the other hand, understanding the disorder requires subtype profiling for individual genders (Sinopoli et al., 2017). One of the frequent subtypes for women is the washing compulsion (Zamanian Azodi et al., 2017). Here, by examining the serum proteome of women with washing subtype of OCD, we aimed to better understand this subtype pathogenicity.

## 2. Methods

### 2.1. Human subjects

In this study, three groups of human serum samples including 20 healthy, 12 OCD (washing subtype), and 12 OCD patients treated with fluoxetine were analyzed and compared in terms of protein expression. In fact, among 35 women with OCD washing type, 12 individuals (sensitive to fluoxetine treatment) were included in the study.

According to inclusion criteria, the healthy women were demographically matched to the patient cases. In addition, these healthy cases were without any history of mental problems in their medical profile or families, or under any psychiatric medicines. Similarly, OCD samples had no history of other diagnosed mental conditions, comorbidities, or under any types of psychiatric treatments as well as any specific medication for any other kinds of diseases. The patients were between 20–30 years old.

For this purpose, Yale-Brown questionnaire was applied for each group (healthy and OCD samples) assessments. The enrollment of moderate OCD samples was based on DSM-V in Taleghani Hospital, Tehran, Iran. Their serum samples were provided prior to fluoxetine prescription.

### 2.2. Sample preparation

Sample collection was done via routine venipuncture. Serum samples were centrifuged at 2000 g and 4°C for 10 min and kept at −80°C for 30 min for clotting at the room temperature.

### 2.3. Proteomic analysis

All 2D-electrophoresis materials were obtained from GE HealthCare Life Sciences^1^ and SERVA Company^2^. Pooling was performed for the two groups individually and the protein extraction was done by 2-DE Clean-Up Kit (GE Healthcare). The procedure was performed with three-time replications for the samples following each group assessment for protein concentration using 2-DE Quant Kit (GE Healthcare). Prior to the first dimension, Isoelectric Focusing (IEF), passive rehydration was applied for 8 hours. The separation based on pI was Bio-Rad PROTEAN IEF Cell, containing 11 cm nonlinear IPG with pH range of 4–7 for 7.5 h at 20°C according to Bio-Rad protocol.

Before the second dimension, a perpetration step was required to equilibrate the IPG strips for 30 min at room temperature for the SDS-PAGE. The HPE FlatTop Tower (horizontal electrophoresis) using 2D HPE™ Double-Gel 12.5 % Kit (Serva Company) performed separation based on MW for about 3.5 h. After electrophoresis, the gels were stained by SERVA HPE™Coomassie® Staining Kit according to the protocol and then scanned using a calibrated GS-800 densitometer (Bio-Rad) scanner (Hasanzadeh, Rezaie-Tavirani, Seyyedi, & Emadi, 2015). Protein expression of two samples were quantified and qualified by Progenesis SameSpots software as an image analyzer.

For expression changes, a value of 1.5-fold was considered and the differentially expressed protein spots were introduced considering (P≤0.05) using 1-way ANOVA analysis. Finally, MALDI TOF-TOF MS analyzed the candidate spots according to the relevant protocol. At the end, evaluation of the extracted peptides was handled by MS and the spectra were submitted to MASCOT^3^ for the purpose of protein identification.

### 2.4. Validation test

Nephelometric quantification (Hitachi Auto Analyzer) of haptoglobin in three OCD samples and six healthy samples was handled following the proteomics as a validation test. Additionally, C-Reactive Protein (CRP), as one of the markers of inflammation in different diseases (Dickerson et al., 2013; Rasmussen et al., 2017), was assessed to provide more information related to our findings. The used method for assessing the concentration of this marker in our patients was turbidimetry (Hitachi Auto Analyzer), in which three samples of healthy and OCD cases were used, individually.

### 2.5. Network analysis

A network of introduced proteins was constructed by Cytoscape V, 3.5.1. software (Shannon et al., 2003) and String database (Szklarczyk et al., 2017). The interaction confidence cut off for the inquired proteins was set at 0.4 and the number of neighbor proteins was selected as 50. The centrality analysis was handled via Network Analyzer which is installed in Cytoscape (Assenov, Ramírez, Schelhorn, Lengauer, & Albrecht, 2007). The two important centrality criteria including degree and betweenness centrality were assessed for network analysis in this study. Cut off for hub and bottleneck was set as >10% of the total nodes with highest degree and betweenness centrality values. Furthermore, the enrichment analysis was handled by DAVID bioinformatics resources 6.8^4^ (Sherman & Lempicki, 2009).

## 3. Results

**Figure 1.**
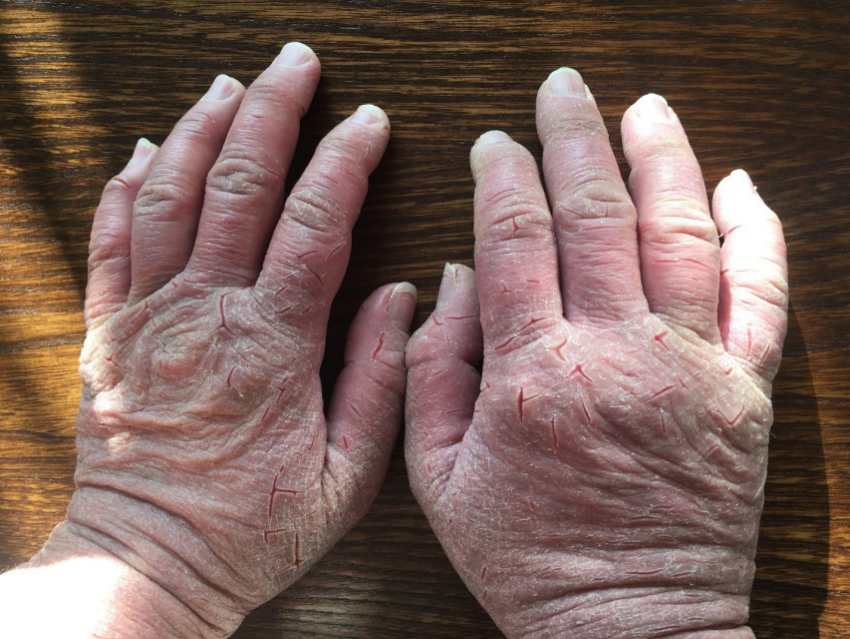
Excessive hand washing in one of our OCD patients caused in red and chapped skin with some bleeding

People with washing subtype of OCD suffer from obsessive thoughts about contamination that compel them to perform ritual manners to temporary alleviate their anxiety (Sarokhani, Sarokhani, Sayehmiri, & Azodi, 2016). Not only this unreasonable behavior is time-consuming, but also it can result in physical injuries (Figure 1).

**Figure 2.**
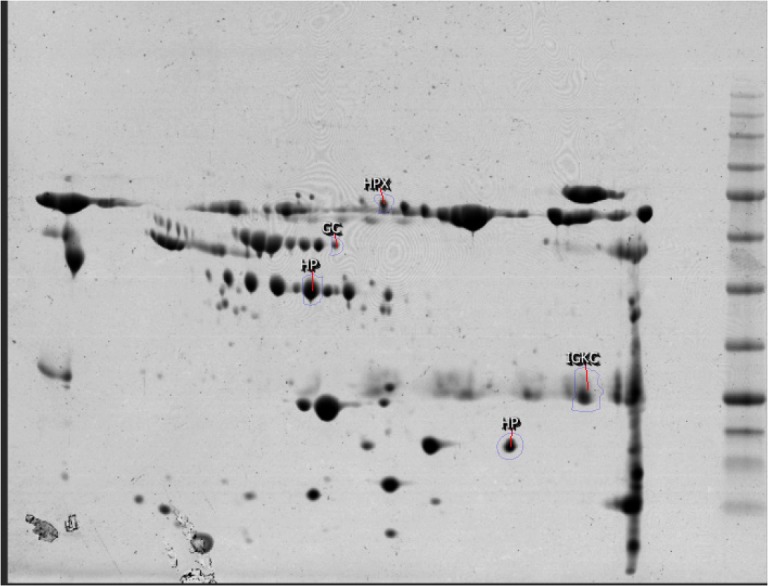
Assigning five identified protein spots via MS d on the OCD patient gel

About 240 protein spots were detected and 5 significantly expressed ones including HP, HPX, GC, and IGKC were identified. These proteins are labeled after mass spectroscopy identification on patient sample 2 DE gel in Figure 2 and the analysis details are presented in Table 1.

**Table 1.** Five identified protein spots expression changes, ranked based on fold change values

**NO**	**S-NO**	**Name**	**MW**	**pI**	**ANOVA (P)**	**Fold**	**ANV (Control)**	**ANV (OCD)**	**Condition**
1	236	HPX	74202	5.65	0.005	1.6	2815.000	4491.952	Up-regulated
2	85	HP	48010	5.32	4.827e-004	1.6	2.071e+004	1.301e+004	Down-regulated
3	118	IGKC	25214	6.71	0.004	2.1	2.1877e+004	1.0471+004	Down-regulated
4	133	HP	17042	6.30	7.008e-004	2.1	1.276e+004	6221.119	Down-regulated
5	59	GC	54526	5.38	0.003	3.5	491.000	1735.079	Up-regulated

S-NO: Spot Number; ANV: Average Normalized Values

**Figure 3.**
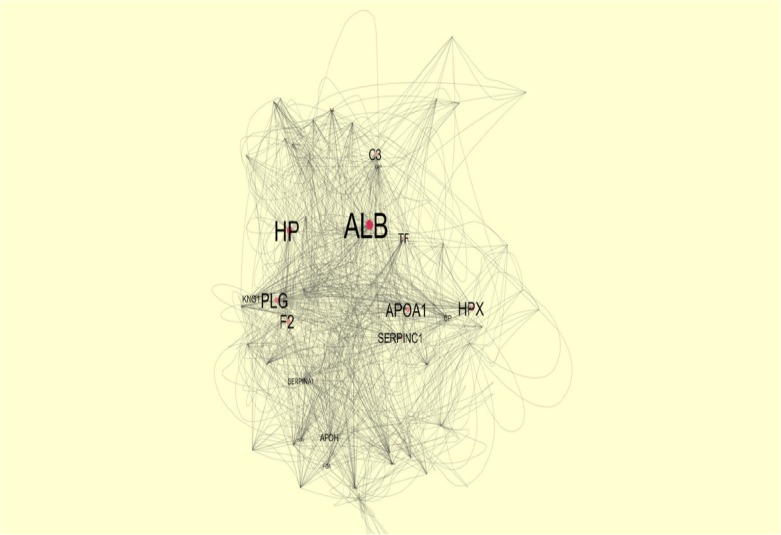
The protein-protein interaction network analysis of the five characterized proteins

MALDI-TOF-TOF MS and MASCOT^5^ analyzed and identified the candidate protein spots (Table 2). To get a better resolution of proteins’ role in a whole interacting system, a network of identified proteins with the close surrounding proteins was mapped (Figure 3). The proteins with the labels are the central nodes in the analyzed network. The bigger the labels, the more central nodes are. The designated cut off for interaction between nodes was set at 0.4.

**Table 2.** The list of identified protein spots with the detailed information obtained from MASCOT (P<0.05)

**Protein Name**	**UniProt Code**	**Protein Seq Coverage (%)**	**Peptide Matches**	**Matching Score**
Ig kappa chain C region	P01834	48	4	380
Hemopexin	P02790	18	7	416
Haptoglobin	P00738	4	7	597
Haptoglobin	P00738	11	6	561
Vitamin D-binding protein	P02774	12	7	434

In Table 3, the centrality analysis of the constructed network of designated proteins are presented. The proteins are ranked based on degree value. The two important centrality parameters including Degree (D) and Betweenness Centrality (BC) are presented. The threshold for hub and bottleneck centralities is assigned as above the 10% of the highest common ranked proteins.

**Table 3.** The centrality analysis of the constructed network of designated proteins

**Row**	**Protein Name**	**Degree**	**Betweenness Centrality**
1	ALB	48	0.05
2	HP	44	0.07
3	PLG	40	0.02
4	HPX	39	0.07
5	APOA1	39	0.02

To understand biology processes related to OCD, enrichment analysis was performed. The associated biological processes of our identified proteins are searched through DAVID bioinformatics v6.8 and shown in Tables 4 and 5. Table 4. The list of biological processes corresponded to each candidate proteins obtained from DAVID bioinformatics V. 6.8.

**Table 4.** Biological processes corresponded to the candidate proteins via DAVID

**Gene Name**	**Protein Name**	**Biological Process**
GC	Vitamin D binding Protein	Vitamin D metabolic process, Vitamin transport
HP	Haptoglobin	Immune system process, receptor-mediated endocytosis, defense response, acute-phase response, positive regulation of cell death, response to the hydrogen peroxide, Defense response to bacterium, negative regulation of oxidoreductase activity, cellular oxidant, detoxification, negative regulation of hydrogen peroxide catabolic process
HPX	Hemopexin	Positive regulation of immunoglobulin production, positive regulation of humoral immune response mediated by circulating immunoglobulin, cellular iron ion hemostasis, receptor-mediated endocytosis, heme transport, viral process, hemoglobin metabolic process, heme metabolic process, positive regulation of tyrosine phosphorylation of Stat1 protein, positive regulation of interferon-gamma-mediated signaling pathway
IGKC	Immunoglobulin kappa constant	Retina homeostasis, proteolysis, receptor-mediated endocytosis, phagocytosis, engulfment, immune response, complement activation, FC-epsilon receptor signaling pathway, FC-gamma receptor signaling pathway involved in phagocytosis, defense response to bacterium, innate immune response, regulation of immune response, B cell receptor signaling pathway, positive regulation of B cell activation

**Table 5.** Chart of linked biological processes to the identified proteins via DAVID

**Term**	**Count**	**%**	**P**	**Benjamini**
Receptor-mediated endocytosis	3	75	3.6E ^−4^	1.2E ^−2^
Defense response to bacterium	2	50	2.6E^−2^	3.6E^−1^

Among four queried proteins, HP, HPX, IGKC are common in receptor-medicated endocytosis and HP and IGKC in defense response to bacterium. Based on designated statistical criteria including threshold count protein per terms:1 and Ease score: 0.1 as the default, and the correction method: Benjamini, GC protein was not resulted in the output. The obtained Ease score for GC=1, The Ease score ranges from 0 to 1.

**Figure 4.**
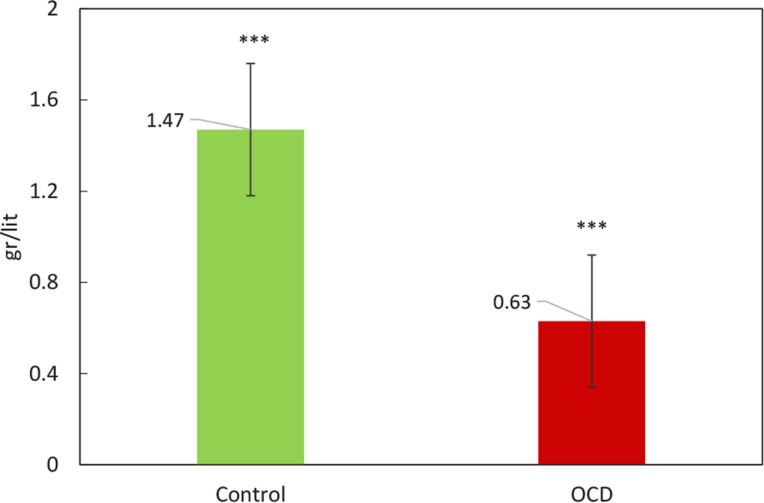
Comparison of levels HP between the control and OCD groups obtained from validation test: Nephelometric quantification The green and red bars denote the average of HP Levels in the control and OCD samples. The error bars signifies standard deviation. The level of HP decreased in OCD samples. ^***^P≤0.001

**Figure 5.**
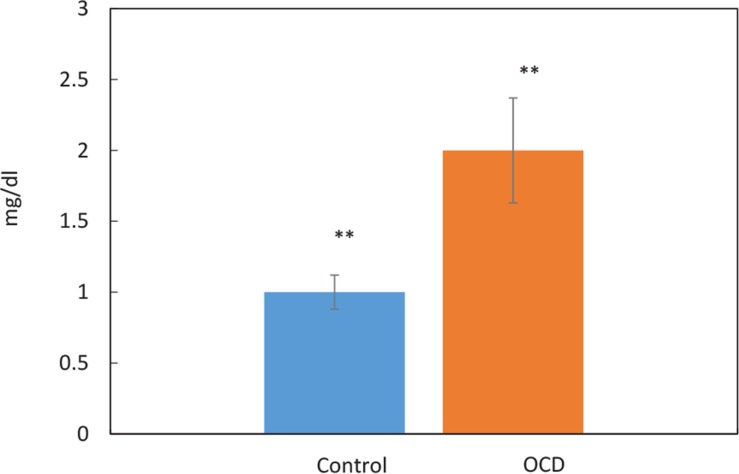
Comparison of CRP levels between the control and OCD groups obtained from validation test: Nephelometric quantification. The blue and red bars indicate the average CRP levels in the control and OCD samples. The error bars signifies standard deviation. The level of CRP increased in OCD samples. ^**^P≤0.01

The pattern of HP Levels changes of healthy and OCD samples via nephelometric test is compared by a bar chart in Figure 4. Further evaluation was conducted to get a better view of OCD mechanisms, based on obtained findings, CRP as one of known inflammation markers was screened in our samples (Figure 5).

## 4. Discussion

Obsessive Compulsive Disorder is a complex neuropsychiatric disorder causing distress in human daily life (Sarokhani et al., 2016). Figure 1 from one of our studied OCD patients, indicates the hardship of OCD washers’ life and what they have to cope with in every single day. Molecular agents that can cause such behavior worth studying. In fact, the molecular aspects of OCD has remained to be evaluated despite many pervious genetic and genome-wide studies (Zamanian Azodi et al., 2017). In this sense, proteomics has proved to be a novel prerequisite towards understanding expressional modification in a disease state (Safaei et al., 2017). Through elucidating fundamental proteins in OCD pathogenicity, the underlying pathways can be identified.

Here, sera proteome of healthy and OCD samples were analyzed and compared. The analysis shows some isoforms of our protein spots that are altered and may have a role in the OCD risk. As indicated in Figure 2, a number of five matched differentially expressed protein spots are shown on the OCD gel, chosen for further analysis by mass spectrometry. The identified proteins are HP, HPX, IGKC, and GC. Of them IGKC, and HP isoforms decreased while GC and HPX isoforms increased in expression levels as tabulated in Table 1.

Further information related to the protein spots identified in our sample are presented in Table 2. To get a better resolution of the role of chosen proteins in an interactome profile, a network of them was constructed in Figure 3. The map analysis expressed that some proteins are prominent in the network integrity and consequently may play important contribution in the disorder. Based on centrality analysis in Table 3, six proteins are shown to have highest values of degree and betweenness, individually known as hub and bottleneck, respectively. Of them, five proteins were common that are assigned as hub-bottlenecks.

HP and HPX as our inquired proteins seem to have more central roles in the network integrity than the other candidate differentially altered proteins. Additionally, the comparison between the expression changes of our found isoforms in OCD sera and in other types of psychiatric disorder denote noteworthy information. In this way, the common and differential mechanisms of these mental conditions can be better understood. All these proteins except IGKC, imply the expression changes in other psychiatric disorders (Seal & Eist, 1966; Maes et al., 1997; Petrov, 2017).

Hemopexin (HPX) as a type 2 acute phase reactant glycoprotein has antioxidant and iron homeostasis activities (Frye et al., 2015). Here, its isoform shows increased levels in our OCD sample that suggests the presence of inflammation in OCD. It is also previously referred that HPX synthesis is induced in OCD condition (Tolosano & Altruda, 2002). In addition, similar behavior of HPX has been reported in other mental disorders such as schizophrenia, mania, and depression (Maes et al., 1997).

However, we were only able to determine one of its isoforms and other ones expressed no significant differential expression changes. Network analysis also implies the importance of this protein in the network strength, which gives more credit to its major position in the disorder. Another significantly elevated protein level in OCD sample is vitamin D binding protein. This protein has fundamental participation in binding to vitamin D (GC). The expression alteration of this protein may have associations with reduced vitamin D levels in OCD patients (Celik, Tas, Varmıs, Tahiroglu, & Avci, 2016; Esnafoğlu & Yaman, 2017).

The increment of GC has been also evident in one recent study of bipolar patients (Petrov, 2017). Immunoglobulin kappa chain C (IGKC), the protein that has been previously studied by our team (Zamanian Azodi et al., 2017), shows lower expression levels in OCD patients. No correlation has been yet reported between this protein and other psychiatric condition to our knowledge. This protein is active in immune response (Lohr et al., 2013).

Two isoforms of haptoglobin were detected that their lower expression levels were determined in OCD patients. Furthermore, our validation study confirms the overall reduced levels of HP in OCD patients (Figure 4). One of the main tasks of HP is antioxidant activity (Tseng, Lin, Huang, Liu, & Mao, 2004), meanwhile it has been known that oxidative stress is one of the active processes in OCD (Behl, Swami, Sircar, Bhatia, & Banerjee, 2010; Shrivastava, Kar, Sharma, Mahdi, & Dalal, 2017). Therefore, the reduced levels of HP in OCD serum may have correlation to this phenomenon. Also, as mentioned earlier, HP is one of the most crucial proteins in an interactome system. All these facts support the indispensable relation of HP in OCD risk. Besides, as it is clear from Tables 4 and 5, enrichment investigation of our identified proteins except for GC, suggests the association of inflammation processes in OCD condition.

Due to the possible correlation of inflammatory agents to OCD, CRP as one of the important markers in the blood, was also screened as a validation test in our patients, individually. The result in Figure 5 shows that the CRP level is elevated in OCD patients’ serum. Moreover, CRP was found to be moderately central in our constructed network. So, the evidence more supports the fact that OCD may be an inflammatory disorder so immunomodulatory therapies may be helpful in this regard (Attwells et al., 2017). Overall, these candidates and their underlying processes may have a potential clinical interest in OCD future treatment.

In conclusion, our findings suggest that some of the blood vital proteins that are responsible in many essential processes in the body may be associated with OCD underlying mechanisms. One of which, HP, validated in this study, may particularly serve as a novel biomarker. Furthermore, this study supports the role of inflammation in OCD risk factor. In view of this new insight to OCD complexity, new therapeutic strategies can be targeted. However, to validate this claim, more investigation is required.

## Ethical Considerations

### Compliance with ethical guidelines

Written informed consent was obtained from all the patients. The ethical code for our study is IR.SBMU. REC.1393.299.

## Footnotes

1. http://www.gelifesciences.com

2. http://www.serva.de

3. http://www.matrixscience.com

4. http://david.niaid.nih.gov

5. http://www.matrixscience.com
